# Case Series: ADHD Phenotype in a Family with NPY Gene Microduplication

**DOI:** 10.3390/jcm15197551

**Published:** 2026-09-29

**Authors:** Paolo Alfieri, Elisa Rolleri, Cristina Caciolo, Antonio Novelli, Giusy Viscogliosi, Silvia Genovese, Francesca Cumbo, Marco Cappa, Deny Menghini, Maria Cristina Digilio, Floriana Costanzo, Stefano Vicari

**Affiliations:** 1 Child and Adolescent Neuropsychiatry Unit, Bambino Gesù Children’s Hospital, IRCCS, 00165 Rome, Italyfloriana.costanzo@opbg.net (F.C.); stefano.vicari@opbg.net (S.V.); 2 Laboratory of Medical Genetics, Translational Cytogenomics Research Unit, Bambino Gesù Children’s Hospital, IRCCS, 00165 Rome, Italy; 3 Unit of Muscular and Neurodegenerative Disorders, Bambino Gesù Children’s Hospital, IRCCS, 00165 Rome, Italy; 4 Research Unit for Innovative Therapies in Endocrinopathies, Bambino Gesù Children’s Hospital, IRCCS, 00165 Rome, Italy; marco.cappa@opbg.net; 5 Genetics and Rare Diseases Research Division, Bambino Gesù Children’s Hospital, IRCCS, 00165 Rome, Italy; 6 Life Sciences and Public Health Department, Università Cattolica del Sacro Cuore, 00168 Rome, Italy

**Keywords:** ADHD, NPY, 7p15.3 microduplication, obesity, memory, neurodevelopmental phenotype

## Abstract

**Background**: Neuropeptide Y is a highly conserved neuropeptide involved in several physiological processes, including stress regulation, appetite control, and cognitive functioning. Emerging evidence suggests that alterations in neuropeptide Y signaling may contribute to neurodevelopmental and behavioural phenotypes. This study describes a family carrying a microduplication involving the neuropeptide Y gene on chromosome 7p15.3, with the aim of exploring its potential association with attention deficit/hyperactivity disorder and specific cognitive vulnerabilities, particularly in the memory domain. **Methods**: Five individuals across three generations of one family carrying the duplication underwent comprehensive clinical and neuropsychological assessment, including standardized measures of intellectual functioning, attention, memory, adaptive skills, and psychopathology. **Results:** A consistent pattern of attentional difficulties was observed across affected individuals, accompanied by internalizing features such as anxiety. Cognitive evaluation revealed selective vulnerabilities in memory domains, involving both verbal and spatial components, across short- and long-term processes. Intellectual functioning ranged from borderline to average levels. In addition, increased body weight and hormonal imbalances were recurrently observed, indicating a possible contribution of neuropeptide Y dysregulation to metabolic phenotype in association with behavioural features. **Conclusions**: These findings support the hypothesis that duplication of the neuropeptide Y gene may be associated with a distinct neurobehavioural profile characterized by attentional difficulties, internalizing symptoms, and selective memory vulnerabilities. The co-occurrence of obesity further highlights the potential role of neuropeptide Y in linking metabolic and neurodevelopmental processes. Although based on a small familial sample, this study contributes to the characterization of genotype–phenotype correlations involving neuropeptide Y and underscores the need for further investigation in larger cohorts.

## 1. Introduction

Neuropeptide Y (NPY), a highly conserved peptide that plays a critical role in the central nervous system (CNS), is one of the most abundant neuropeptides in the brain. It is predominantly located in regions such as the hypothalamus, amygdala, hippocampus, and neocortex [1,2]. NPY exerts its effects through interactions with several receptor subtypes, known as NPY receptors. The primary NPY receptors in the CNS are Y1, Y2, Y4, Y5, and Y6, each with distinct, although sometimes overlapping, roles and distribution patterns [3]. The Y1 and Y2 receptors, in particular, are primarily expressed on the post- and presynaptic membranes, respectively. Y1 is mainly found in the cerebral cortex and in the hypothalamus [4,5], while Y2 is also highly concentrated in the hypothalamus [5,6], as well as in the amygdala, hippocampus, and basal ganglia [7,8,9].

Y1 receptors are implicated in modulating anxiolytic effects, influencing the release of corticotropin-releasing hormone (CRH) [10], whereas Y2 receptors often act as autoreceptors, inhibiting the release of NPY and other neurotransmitters to modulate synaptic transmission and often having opposite effects to Y1 receptors in the regulation of emotional states, with an anxiogenic effect [11]. Both receptors are also involved in cognitive functions, particularly in memory and learning [12,13].

The regulation of Y4 and Y5 receptors are primarily implicated in regulating energy balance, with Y4 reducing appetite and Y5 increasing it. Dysregulation of these receptors has been linked to obesity and metabolic disorders [14,15].

NPY, through its receptors, acts on multiple levels, from regulating neurogenesis [16] to modulation of the excitatory/inhibitory balance of different networks [8]. Combining its broad distribution with its wide range of receptor subtypes, NPY modulates numerous physiological and pathophysiological processes, including stress response, appetite regulation, circadian rhythms, reward regulation and mood processing. Moreover, its role in network excitability and homeostasis suggests a potential involvement in the epileptogenic process and its possible therapeutic applications in antiepileptic treatment [17].

NPY is not only present in the CNS, but it is also expressed in the peripheral nervous system, influencing nociception [18], as well as in the heart muscle [19], regulating endocardial endothelial cell function. NPY also acts as an appetite-increasing hormone, and its overexpression has been associated with increased risk for metabolic syndrome and obesity [20].

The NPY gene is located on the short arm of chromosome 7 (7p15.3). The expression of the gene is regulated by various factors, including hormonal signals, stress, and circadian rhythms. Gene activity can be influenced by both environmental factors and genetic predispositions, and variants in the NPY gene can affect peptide expression and function. These variants and have been associated with various disorders and conditions [21,22].

NPY gene duplication has been correlated with an increase of its plasma concentration and a change in its concentration at the encephalic/plasma level, either in excess or in deficiency. The altered levels of NPY are related to the onset of psychopathological processes [23,24].

A correlation between NPY duplication and Attention Deficit Hyperactivity Disorder (ADHD) has been suggested [25,26], even though no definitive evidence has been reported. In particular, NPY and its receptors are involved in some of the neural hubs and pathways that are linked to ADHD, such as dopamine activity in the prefrontal cortex [27]. Indeed, the absence of the Y2 receptors has been associated with reduced attention and increased impulsivity [28], as their presence in the hippocampus and amygdala may be involved in sustained attention and effective performance. In addition, the Y2 receptors have a role in many disorders that are often comorbid with ADHD, such as substance addiction, with NPY levels in the nucleus accumbens being associated with mechanisms of reward and reinforcement [9].

In the present report, we describe and evaluate five individuals from three generations of the same family (Figure 1), all carrying the same NPY gene microduplication, who exhibit a characteristic behavioural and functioning profile.

## 2. Methods

Five individuals from a single family across three generations (Figure 1) were evaluated following the identification of a 511 kb microduplication at 7p15.3 involving the NPY gene and part of PALS2: two sisters (Cases A and B), their father (F), paternal aunt (N), and paternal grandfather (G). The mother of Cases A and B tested negative.

Cases A, B, and F underwent the most comprehensive assessments, including cognitive and memory evaluations, whereas N and G completed only psychopathological and adaptive behaviour measures. The two sisters were assessed longitudinally (Case A: four evaluations over 7 years at ages 9, 11, 12, and 16; Case B: three evaluations over 5 years at ages 19, 20, and 24) and were additionally monitored for growth parameters (weight and height) as well as metabolic and endocrinological profiles (including hormonal measures).

The adult relatives each completed a single evaluation (F at age 46, N at age 44, and G at age 70).

## 3. Results

### 3.1. Case A

A was referred to our Centre at age 8 because of attention difficulties in the school setting. Her prenatal and perinatal history, as well as her developmental milestones, were reported as unremarkable. However, behavioural difficulties began to emerge during her preschool years, where she exhibited intense agitation and difficulty separating from her mother, even to the point of vomiting. Her preschool teachers also noted challenges in her ability to maintain focus for sustained periods.

As A progressed to primary school, behavioural and mood changes were observed by her parents, including increased irritability, low self-esteem, and phobic symptoms. She also exhibited disorganized and excessive eating behaviours, which eventually led to obesity and hepatic steatosis by age 7. Her difficulties regarding concentration were noted by her teachers, along with issues in completing assignments.

A’s first neuropsychological evaluation occurred at age 9, when her cognitive profile was assessed (WISC-IV). The results were heterogenous, showing a discrepancy of over 12 points between indices, all falling within the average range for her age, except the working memory index (WMI = 70).

A psychopathological evaluation (K-SADS-PL) as well as an assessment of her executive functions and learning abilities were also conducted (see Table 1), leading to a diagnosis of ADHD associated with generalized anxiety disorder and learning disorder in reading and writing. At this time genetic testing through array CGH analysis was conducted, revealing the aforementioned NPY microduplication (arr[GRCh37] 7p15.3(24215881-24727358)x3), and leading to genetic testing of the other family members. The microduplication included a part of the PALS2 gene, which does not appear to be involved in neurological development or metabolic functions (OMIM* 606959).

A had three further assessments over a period of 7 years: at ages 11, 12 and 16. Although her IQ was within the normal range in the first two evaluations, her cognitive testing, via WAIS-IV, at age 16 revealed borderline intellectual functioning (See Table 2). The WMI consistently presented as the lowest score among the indices, with a maximum discrepancy of over 25 points in respect to the other indices. Moreover, both verbal memory and spatial memory, tested at age 11 and 16, were affected to varying degrees. Adaptive behaviour was assessed (ABAS II) and ranged from average to the lower end of the normal range across time points (See Table 3). During the follow-up visits, fluctuating levels of anxiety symptoms were reported, with periods of wellness alternating with episodes of increased distress and functional impairment. Her SLC-90-R scores at last evaluation, aged 16, was altered in multiple areas and reached clinical significance in the obsessive-compulsive and psychoticism dimensions.

### 3.2. Case B

B, A’s older sister, shares the same genetic microduplication as A and was first evaluated at the age of 18, at which time she did not report any significant behavioural difficulties. However, in a self-administered psychometric questionnaire (SCL-90-R), B achieved clinically significant scores in areas related to paranoid ideation, obsessive-compulsive symptoms, and depression. B also reported moderate difficulties concerning concentration and memory, which had led to her repeating a year in high school.

Cognitive testing (WAIS-IV) of B at age 19 revealed a full-scale IQ (90) in the normal range but a significant discrepancy between the WMI (72), which was below average, and the other indices. This profile was similar to the A’s profile. Also like her sister, B exhibited difficulties in verbal memory, particularly short-term retention, but her spatial memory remained intact (Figure 2).

She had two more follow-up assessments at ages 20 and 24, which showed fluctuating anxiety symptoms and ongoing challenges with concentration and memory; however, overall, B’s cognitive and adaptive performance remained consistent with the first evaluation.

### 3.3. Other Family Members

The father, F, also carried the NPY microduplication and had a clinical history marked by difficulties with concentration and memory, particularly in academic settings. His cognitive profile via WAIS IV, assessed at age 46, was overall in the normal range, with WMI as the lowest score in his profile (Table 2). F’s psychopathological profile, evaluated with the SCL-90-R, indicated primarily sleep difficulties, with no significant symptoms reported in other areas. His adaptive functioning (ABAS II) was found to be in the normal range.

Their paternal aunt (N)’s SCL-90 questionnaire results indicated clinically significant scores in all the psychopathological areas investigated, except for sleeping problems. She did not report any significant functional impairments at the time of her assessment.

Their paternal grandfather, G, although reporting some difficulties with concentration and memory, did not present any major psychopathological symptoms according to the SCL-90-R. G’s clinical history and testing did not indicate any functional impairments related to his genetic condition. Figure 3 illustrates the frequency of each symptom among family members. 

Regarding non-carrier family members, the mother of Cases A and B, who tested negative for the NPY microduplication, did not report current or previous symptoms of inattention or hyperactivity/impulsivity during clinical and anamnestic assessment, nor a history of obesity. However, no standardized neuropsychological assessment was available; therefore, these observations should be interpreted with caution.

### 3.4. Memory Assessment

All family members reported subjective memory difficulties. Formal memory assessment was performed in the two sisters and their father using age-appropriate neuropsychological tests investigating both short- and long-term memory in the spatial and verbal domains. Spatial memory is defined as the capability of orientating in space and of remembering different locations and the spatial relations between objects. Verbal memory involves the ability to encode, store, and recall information that is presented in a verbal format, such as words, sentences or stories.

We found that, while the verbal memory, especially short-term, was impaired to various degrees, visuo-spatial memory was unaffected, and all three subjects reached normal range scores. For ease of visual interpretation, the results are summarized in Figure 2 using a three-color coding system.

Case A showed stable visuospatial abilities alongside persistent verbal short-term deficits and a decline in verbal long-term memory at follow-up. She was first evaluated at 11 years of age, and her verbal short-term memory was markedly deficient, while verbal long-term memory was within the normal range despite reduced performance on the verbal learning task. Short-term spatial memory was in the normal range, as was long-term spatial memory. When she was re-evaluated at age 16, she achieved similar results, except for long-term memory, which was below average.

Case B was first evaluated at 18 years of age. Verbal short-term memory was markedly deficient, while verbal long-term memory was at the lower limit of the normal range. At 24 years, verbal short-term memory improved to the lower limit of the normal range, while verbal long-term memory remained at the lower limit of the normal range. Short-term spatial memory and long-term visuospatial memory were within the normal range at both time points.

The father exhibited a similar, though milder, profile, with verbal memory, both short- and long–term, at the lower limit of the normal range and preserved visuospatial performance overall.

Overall, these findings suggest a domain-specific memory profile, characterized by relative impairment in verbal processes alongside sparing of visuospatial memory, consistent across affected family members.

### 3.5. Endocrinology Evaluation

Clinical and endocrinological assessments of the two sisters highlighted a shared pattern of increased body mass index (BMI) and emerging metabolic and hormonal abnormalities. A history of obesity was also reported in the father; however, no formal clinical or laboratory evaluation was available.

Case A was referred for an endocrinological evaluation at 7 years and 3 months of age. Perinatal history revealed a birth weight of 3780 g and a length of 50 cm. Past medical history was notable for hepatic steatosis.

At the time of her first endocrinological evaluation, her height was 128.3 cm (0.84 SD), weight 45.8 kg (2.45 SD), and BMI 28.8 kg/m^2^ (2.45 SD). Hypercortisolism was excluded by measuring 24 h urinary free cortisol using LC-MS/MS.

At follow-up at 7 years and 9 months, her height was 131.0 cm (1.56 SD), weight 48.1 kg (2.73 SD), and BMI 28 kg/m^2^ (2.49 SD). Laboratory evaluation showed a normal lipid profile (total cholesterol, HDL, LDL). Apolipoprotein (Apo) A1 was 175 mg/dL (reference range 55–135), Apolipoprotein B 65 mg/dL (55–135), and the ApoB/ApoA1 ratio was 0.37 (reference range 0.65–0.95). The Homeostatic Model Assessment for Insulin Resistance (HOMA-IR) index was 1.98 (normal < 2.5).

Menarche occurred at 10 years and 9 months. At 12 years and 3 months, her height was 160 cm (0.95 SD), weight 103 kg (3.58 SD), and BMI 40.2 kg/m^2^ (3.39 SD). During follow-up, she did not adhere to nutritional recommendations. At 16 years and 1 month, her height was 161 cm (−0.16 SD), weight 108 kg (3.69 SD), and BMI 41.7 kg/m^2^ (3.34 SD).

Hormonal evaluation revealed mild hyperinsulinemia (HOMA-IR index 4.19; normal < 2.5) and mild hyperandrogenism, with Δ4-androstenedione 2.4 ng/mL (reference range 0.27–1.63), FSH 3.5 mU/mL, LH 11.7 mU/mL, and an LH/FSH ratio of 3.0 (normal < 1). Overall, these findings indicated severe obesity associated with biochemical hyperandrogenism, consistent with early polycystic ovary syndrome (PCOS).

The older sister, case B, presented neonatal overgrowth, with a birth weight of 4080 g and length of 55 cm. Menarche occurred at 12 years of age.

At the time of evaluation (23 years old), her height was 163 cm (0.06 SD), weight 79 kg (2.11 SD), and BMI 29.7 kg/m^2^ (2.09 SD). Hormonal evaluation showed elevated testosterone (96.7 ng/dL; reference range 40–68) and Δ4-androstenedione (2.9 ng/mL; reference range 0.27–1.63). Gonadotropins were FSH 3.3 mU/mL and LH 10.7 mU/mL, with an LH/FSH ratio of 3.2 (normal < 1). Leptin levels were 20 ng/mL. Hypercortisolism was excluded by 24 h urinary free cortisol measurement using LC-MS/MS.

These findings indicate overweight status associated with biochemical hyperandrogenism and ectopic hepatic fat deposition, which might be consistent with an early diagnosis of PCOS.

## 4. Discussion

Our findings suggest that the NPY microduplication may contribute to a distinct neuropsychological phenotype, including features of ADHD, inattentive symptoms, internalizing psychopathology, and metabolic disturbances affecting the BMI.

All individuals reported attention deficits and verbal memory difficulties, and while the cognitive evaluation showed normal full-scale IQ, verbal working memory difficulties were prominent, consistent with the cognitive deficits commonly reported in ADHD populations [43].

Although NPY levels were not assessed in the present family, limiting direct assessment of the relationship between the gene duplication and NPY expression, previous human evidence provides biological plausibility for a gene-dosage effect: Lesch et al. reported increased plasma NPY concentrations and altered reward- and emotion-related brain activation in carriers of a 7p15.2–p15.3 duplication encompassing NPY [44].

Adaptive behaviour assessment indicated that most family members scored within the normal range overall. However, examination of the subscales comprising the adaptive functioning profile revealed a relative weakness in the conceptual domain. Specifically, scores in this domain fell within the clinical or subclinical range (i.e., the lower limit of the normal range) for two participants and represented the lowest domain score for three of the five participants. Difficulties in the conceptual domain, which includes Communication, Functional Academics, and Self-Direction, are consistent with the verbal working memory deficits observed in our sample and align with the existing literature indicating that individuals with ADHD frequently experience impairments in adaptive functioning, especially in executive functioning and academic performance [45,46].

Furthermore, a trend towards higher-than-average scores for depression and obsessive-compulsive symptoms was observed in the female family members (A, B, N), consistent with the proposed involvement of the NPY system in the regulation of mood and emotional functioning [47].

Another significant observation was the increased BMI in the majority of the affected family members, including obesity in A and F and overweight in B. These findings align with previous studies that have shown a link between NPY gene activity and increased appetite and body weight [20].

Moreover, the youngest family member (A) was diagnosed with hepatic steatosis at age 7, further supporting the hypothesis that NPY gene duplication may predispose individuals to metabolic syndromes. This is particularly relevant in the context of ADHD, as patients with ADHD, particularly those with the combined subtype, are at a higher risk for obesity and related metabolic disorders [48,49]. Additionally, the paternal side of the family reported a history of type 2 diabetes, suggesting a potential familial predisposition to metabolic disturbances.

One of the most intriguing findings in this study was the differential impact on spatial memory and verbal memory. While spatial memory was largely unaffected, verbal memory, particularly short-term verbal memory, was significantly compromised across all tested individuals. This finding is consistent with preclinical studies that have shown a selective effect of NPY on different memory domains. Indeed, animal studies, designed to address the possible effects of different NPY levels on memory and stress, showed that increased levels of NPY, often induced by stress exposure, can have complex effects on memory. Specifically, stress-induced NPY increases, particularly through Y1R activation, have been shown to enhance spatial memory retention and retrieval, while they may impair certain types of non-spatial memory, such as verbal memory or implicit memory retention, depending on the context and the brain regions involved [13,50].

ADHD is traditionally categorized as a distinct neurodevelopmental disorder; however, it is increasingly recognized that it represents a heterogeneous condition characterized by a constellation of symptoms. These symptoms can manifest in different combinations and degrees of severity across individuals. As a result, ADHD is not a monolithic disorder but rather comprises multiple subtypes with varying degrees of emotional and cognitive impairments [51]. The absence of hyperactivity in this family stands in contrast to the more typical presentation of ADHD. This observation raises the possibility that NPY microduplication may be associated with a specific subtype of ADHD that involves attention deficits and internalizing symptoms without the classic hyperactive component.

The findings from this case series highlight the importance of genetic factors in shaping the clinical presentation of ADHD. Understanding how specific genetic mutations like the NPY gene microduplication may contribute to a unique ADHD phenotype may open new avenues for targeted interventions. In particular, both sisters presented obesity of varying degrees and hyperandrogenism. Their hormonal profiles are consistent with progression toward PCOS, a condition frequently observed during adolescence and adulthood in individuals with obesity. Metabolic health, particularly addressing obesity and associated conditions such as hepatic steatosis and type 2 diabetes, should be considered in the management of ADHD, especially in individuals with NPY-related genetic alterations. This integrated approach could help mitigate the risk of comorbid metabolic and psychiatric conditions that are prevalent in ADHD patients.

However, we are aware of the limitations of our study, specifically, all the individuals described belong to a single family and therefore share both genetic background and environmental factors. Consequently, the contribution of other co-inherited variants, linkage disequilibrium, or shared environmental influences cannot be excluded, and no causal relationship between the NPY variant and the observed ADHD-related phenotype can be established. Rather, our findings should be considered a preliminary clinical observation that supports further investigation of NPY as a potential candidate biomarker in larger, independent cohorts and through functional studies examining its association with the ADHD phenotype, as well as its potential influence on cognitive functioning, mood regulation, and metabolic health.

## Figures and Tables

**Figure 1:**
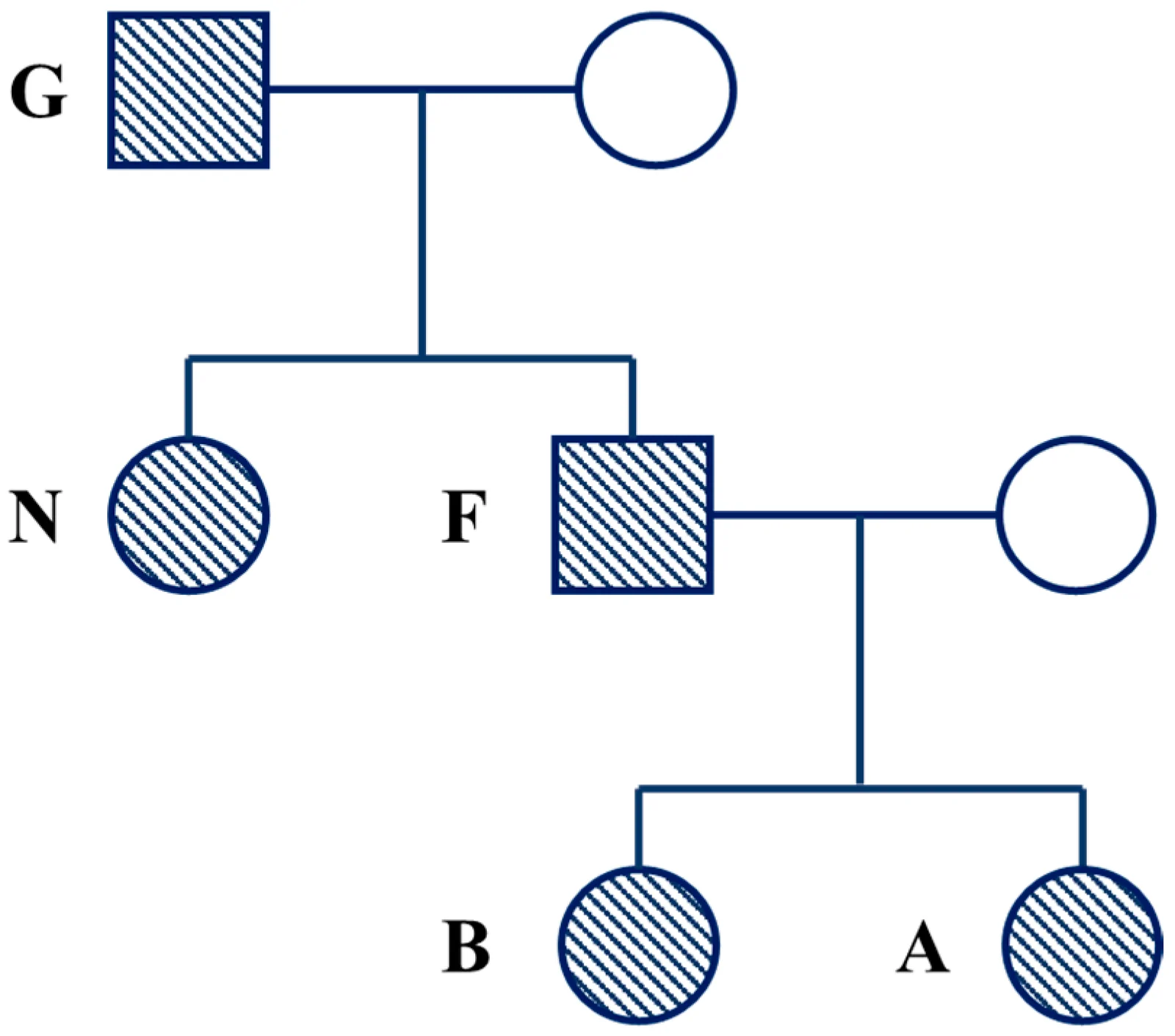
Pedigree of the three-generation family carrying the NPY microduplication at 7p15.3. Squares represent males and circles represent females. Shaded symbols indicate individuals carrying the duplication; unshaded symbols represent non-carriers. The two sisters (Case A and Case B) are shown. Their father (F), aunt (N) and grandfather (G) also carry the duplication.

**Figure 2:**
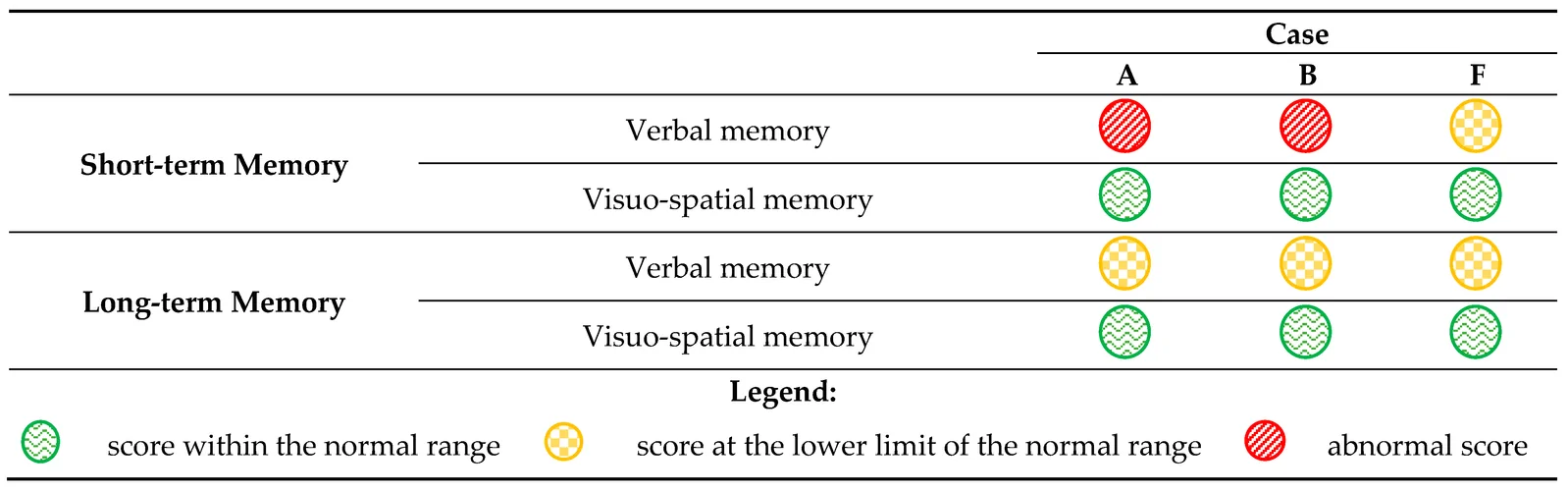
Summary of memory performance in cases A, B, and F.

**Figure 3:**
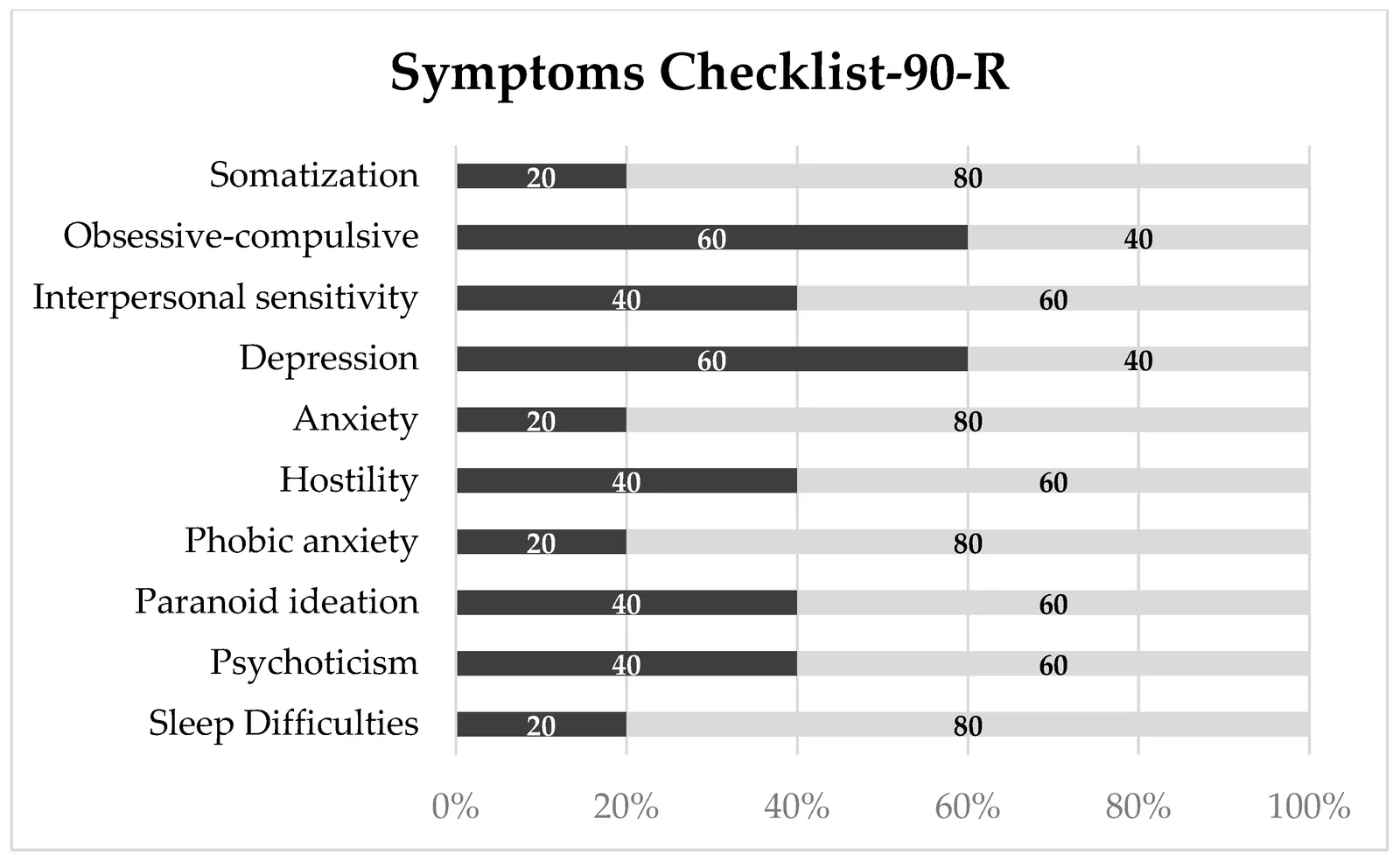
The frequency of each of the symptoms within the family as evaluated through the Symptom Checklist-90-R (SCL-90-R), a self-report questionnaire designed to evaluate a broad range of psychological problems. For each of the ten primary dimensions, values represent the percentage of family members reaching a clinically significant score (with 100% corresponding to all individuals reporting the symptom).

**Table 1:** Assessment battery.

	Case				
	A	B	F	N	G
**Cognitive abilities**					
Wechsler Intelligence Scale for Children-IV (WISC-IV) [29]	x				
Wechsler Adult Intelligence Scale-IV (WAIS-IV) [30]	x	x	x		
**Memory**					
**Short-term verbal:** Digit forward span [30,31,32]; PROMEA low-frequency bisylabic words test [33]	x	x	x		
**Short-term visuospatial:** Corsi Block-Tapping Test [30,32], TEMA spatial memory test [31], PROMEA spatial span test [33]	x	x	x		
**Long-term verbal:** Rey Auditory Verbal Learning Test [34], PROMEA verbal learning test [33], NEPSY-II word list memory test [35]	x	x	x		
**Long-term visuospatial:** Rey–Osterrieth complex figure [36]; PROMEA spatial learning test [33]	x	x	x		
**Executive functions/attention**					
Behavior Rating Inventory of Executive Function, Second Edition (BRIEF-2) [37]	x				
**Psychopathological profile**					
Conners’ Parent Rating Scale–Revised (CPRS-R) and Conners’ Teacher Rating Scale–Revised (CTRS-R) [38]	x				
Kiddie Schedule for Affective Disorders and Schizophrenia for School-Age Children–Present and Lifetime Version (K-SADS-PL) [39]	x	x			
Symptom Checklist-90-R (SCL-90-R) [40]	x	x	x	x	x
Child Behavior Checklist for Ages 6–18 and Youth Self-Report for Ages 11–18 [41]	x	x			
**Adaptive behavior**					
Adaptive Behavior Assessment System—second edition (ABAS-II) [42]	x	x	x	x	x

Tests that were administered are marked with an x.

**Table 2:** Cognitive profile of the analysed individuals at last evaluation.

		Case		
		A	B	F
	Age at evaluation (years)	16	24	46
**WAIS-IV**	Verbal Comprehension Index	94	106	103
	Perceptual Organization Index	79	98	90
	Working Memory Index	69	63	87
	Processing Speed Index	95	122	96
	Full Scale IQ	80	98	94

**Table 3:** Adaptive behaviour of the five analysed individuals.

		Case				
		A	B	F	N	G
	**Age at evaluation (years)**	11	19	46	44	70
**ABAS-II**	Conceptual Domain	70	92	72	97	113
	Socialization Domain	78	94	82	97	95
	Daily Living Skills	98	95	84	117	106
	General Adaptive Composite	83	94	80	103	107

## Data Availability

Data supporting the conclusions of this study will be available from the corresponding author upon reasonable request.

## Abbreviations

ABAS-II	Adaptive Behavior Assessment System, Second Edition
ADHD	Attention Deficit Hyperactivity Disorder
ApoA1	Apolipoprotein A1
ApoB	Apolipoprotein B
BMI	Body Mass Index
CBCL	Child Behavior Checklist
CGH	Comparative Genomic Hybridization
CNS	Central Nervous System
CRH	Corticotropin-Releasing Hormone
FSH	Follicle-Stimulating Hormone
HOMA-IR index	Homeostasis Model Assessment Index
IQ	Intelligence Quotient
K-SADS-PL	Kiddie Schedule for Affective Disorders and Schizophrenia for School-Age Children–Present and Lifetime Version
LC-MS/MS	Liquid Chromatography–Tandem Mass Spectrometry
LH	Luteinizing Hormone
NEPSY-II	A Developmental NEuroPSYchological Assessment, Second Edition
NPY	Neuropeptide Y
PCOS	Polycystic Ovary Syndrome
PROMEA	Prove di Memoria e Apprendimento
SCL-90-R	Symptom Checklist-90—Revised
TEMA	Test of Memory and Learning (Italian adaptation context)
WAIS-IV	Wechsler Adult Intelligence Scale, Fourth Edition
WISC-IV	Wechsler Intelligence Scale for Children, Fourth Edition
WMI	Working Memory Index
